# Wireless asymmetric umpolung electrosynthesis†

**DOI:** 10.1039/d4cc02406k

**Published:** 2024-07-01

**Authors:** Sara Grecchi, Bartlomiej Bonczak, Filippo Malacarne, Gerardo Salinas, Roberto Cirilli, Serena Arnaboldi

**Affiliations:** a Dip. Di Chimica, Univ. degli Studi di Milano Milan Italy serena.arnaboldi@unimi.it; b Univ. Bordeaux, CNRS, Bordeaux INP, ISM UMR 5255 F-33607 Pessac France; c Istituto Superiore di Sanità, Centro Nazionale per il Controllo e la Valutazione dei Farmaci Rome Italy

## Abstract

Electroorganic synthesis has become an exciting tool for the asymmetric conversion of pro-chiral compounds. Herein, we introduced a wireless methodology based on bipolar electrochemistry in synergy with the enantioselective capabilities of inherently chiral oligomers to induce an umpolung chirality transfer. This was exemplified by the electro-conversion of a racemic mixture of lansoprazole to an *enantio*-enriched solution of a single antipode.

## Abstract 2

Electroorganic synthesis has become an exciting tool for the asymmetric conversion of pro-chiral compounds.

## Graphical abstract

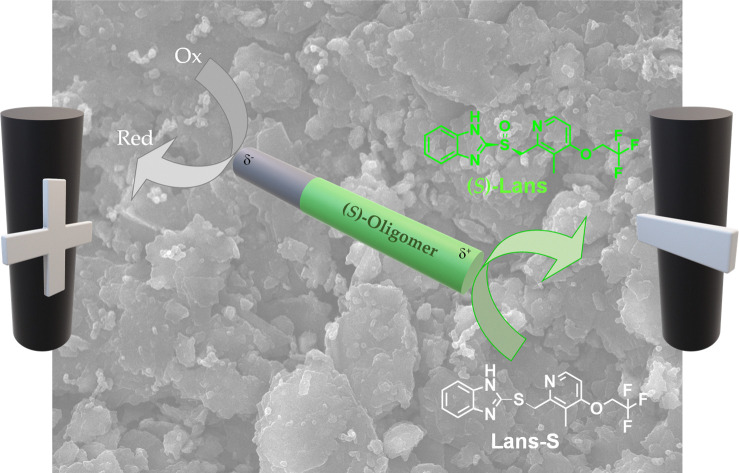

Recently, electroorganic synthesis has found a so-called renaissance due to the possible use of electricity as a green and cheap reagent.^1^ Among the multiple synthetic schemes, the asymmetric electroconversion of pro-chiral compounds has gained considerable attention.^2^ In particular, developing novel synthetic routes to chiral molecules with high enantiomeric excess (ee) values is paramount for the pharmaceutical industry. Different approaches involving chiral-mediated reactions or imprinted metallic electrodes have been developed.^3^ An interesting alternative is using electrodes modified with inherently chiral oligomers, in which the stereogenic and electroactive elements coincide within the polymeric backbone.^4^ Thus, favorable or unfavorable diastereomeric interactions between the chiral surface and the antipodes of an analyte are induced. Such energetic differentiation is expressed by relatively significant thermodynamic potential differences between the two enantiomers. Moreover, it has been demonstrated that the enantioselectivity is not related to complexation host–guest interactions or is exclusively limited to the presence of an external electric field.^5^ Taking advantage of such outstanding properties makes it possible to transfer chirality across different length scales to develop optical and dynamic readouts of chiral information.^6^ In particular, these systems benefit from wireless asymmetric polarization triggered by bipolar electrochemistry (BE).^7^ Briefly, by applying a high enough electric field (*ε*), a polarization potential difference (Δ*V*) across a conducting object or a bipolar electrode (BPE) is induced. When Δ*V* overcomes the thermodynamic threshold potential (Δ*V*_min_), redox reactions are triggered at both extremities of the BPE for a given set of electroactive species. BE has gained considerable attention as an unconventional approach for organic electrosynthesis due to multiple advantages, *i.e.* its intrinsic wireless feature, the possible use of BPE arrays, and easy supporting electrolyte removal.^8^ However, the development of asymmetric transformations based on BE is still a challenge.

This work uses the synergy between BE and inherent chiral oligomers to transfer chirality *via* a wireless umpolung methodology. For this purpose, the reduction/oxidation of lansoprazole (LAN) was selected as the model reaction. LAN presents two additional redox states (Scheme 1a): the lansoprazole sulfide (LAN-S, reduced state) and lansoprazole sulfone (LAN-SO, oxidized state).^9^ Moreover, due to the presence of the lone electron pair on the sulfur atom, LAN exhibits two enantiomeric structures, namely levolansoprazole (l-LAN) and dexlansoprazole (d-LAN). The latter is the active ingredient of the commercial drug commonly used for treating digestive illness, *i.e.*, ulcers or reflux. However, the racemic mixture of LAN is produced during its classic synthesis. In the here presented approach, the wireless electrochemical transfer of chirality occurs in a two-step procedure. At first, the non-chiral reduction of a racemic mixture of LAN occurring at the cathode of a graphite rod, acting as the BPE, is carried out to produce LAN-S (Scheme 1b top). Afterward, the enantioselective oxidation of LAN-S at the anode of a graphite BPE modified with 2-([2,2'-bithiophen]-5-yl)-3-(2-([2,2′-bithiophen]-5-yl)benzo[*b*]thiophen-3-yl)-*N*-methylindole (Scheme 1c), nicknamed BTIndT_4_, was performed (Scheme 1b bottom). This wireless electrosynthesis reaction sequence facilitates the umpolung transfer of chirality (Scheme 1a). Nonetheless, according to the principles of BE, to trigger the reactions of interest, a counterpart redox process occurs at the opposite extremity of the BPE (Scheme 1b red arrows). Under these conditions, it is possible to assume that such reactions involve the oxidation and reduction of the electrolytic media.

**Scheme 1:**
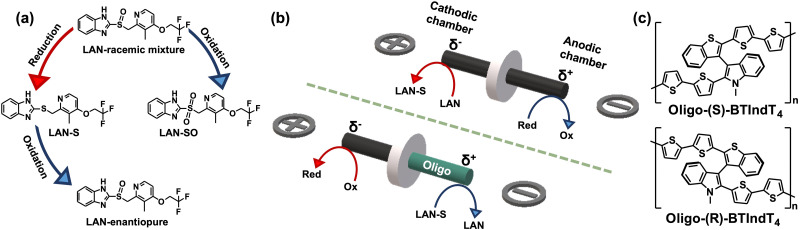
(a) Chemical structures and possible sequence of electrochemical reactions involving the LAN, LAN-S and LAN-SO. (b) Schematic illustration of the bipolar setup used for (top) the wireless reduction of LAN and (bottom) the enantioselective oxidation LAN-S with a representation of the asymmetric polarization and the associated electrochemical reactions. (c) Chemical structures of enantiopure oligo-(*R*)- and oligo-(*S*)-BTIndT_4_ (indicated in the scheme).

BTIndT_4_ was chemically synthesized in our laboratory following a sequence of reactions previously reported.^10^ The potentiodynamic characterization of this monomer results in two oxidation peaks and one reduction signal, taking place at the surface of a graphite rod (*Ø* = 0.3 cm), acting as a working electrode (Fig. S1a, ESI†). The first redox waves (*I*_a_/*I*_c_) are associated with the oxidation of the indole moiety, whereas peak IIa corresponds to the irreversible oxidation of the thiophene groups. However, due to the high conjugation of BTIndT_4_, which stabilizes the produced radical cations, potential values above 1.1 V *vs*. Ag/AgCl are required to trigger the oligomerization. The electro-oligomerization of BTIndT_4_ was performed on the surface of a graphite rod in a 0.75 mM monomer, ACN + 0.1 M tetrabutylammonium perchlorate (TBAP) solution. As can be seen, the electrochemical growth of the monomer exhibits a current increase as a function of the number of cycles, characteristic of the electrodeposition of the π-conjugated polymers (Fig. S1b, ESI†). The enantioselective test of LAN was carried out with such modified electrodes. The potentiodynamic characterization of d-LAN results in an oxidation peak around 1 V *vs*. Ag/AgCl when using the oligo-(*R*)-BTIndT_4_ modified electrode. In contrast, the same oxidation peak occurs at 1.45 V *vs*. Ag/AgCl with the opposite oligomer configuration (Fig. S2, ESI†). Interestingly, the oxidation of d-LAN at the surface of a graphite electrode occurs at a similar potential value, indicating that the unfavorable diastereomeric interaction leads to an inhibition of the electron transfer.

After this set of experiments corroborating the enantioselectivity of the oligomers of BTIndT_4_, the umpolung transfer of chirality was tested. As stated above, the first step is the wireless non-chiral reduction of the racemic mixture of the enantiomers of LAN to LAN-S. For this, a 3 cm long graphite rod was immobilized by a silicon ring, acting as a separator, at the center of a closed bipolar cell containing a 5 mM lithium perchlorate (LiClO_4_) ACN solution. Optical pictures of the complete bipolar electrochemical setup can be found in the ESI† (Fig. S3). A close configuration was chosen to avoid the reciprocal contamination of the solutions and the formation of LAN-S on the cathodic feeder electrode. In addition, as it is well-known, the closed BE setup minimizes the loss of current associated with the migration of ions across the solution. Thus, only the faradaic current passes across the BPE.^11^ Furthermore, due to the geometry of the BPE, at the time scale of this experiment, it is possible to assume that cylindrical diffusion is the principal mass transport limitation. A 10 mM LAN solution was reduced on the cathodic chamber by applying a constant *ε* value (2.2 V cm^−1^) between two feeder electrodes for 120 min. After this, the cathodic solution was analyzed using high-performance liquid chromatography (HPLC). The chromatographic analysis of the individual compounds was properly carried out to compare the HPLC signals obtained after the electrosynthetic steps. These chromatograms showed that the molecule with the lowest retention time is the LAN-SO, followed by the two enantiomers of LAN, and finally, the reduced form, LAN-S (Fig. S4, ESI†). Therefore, after the reduction reaction, an HPLC signal with a retention time between 10 min and 12 min is expected. Before the electrochemical reduction of LAN, only two well-defined signals were obtained, characteristic of d- and l-LAN, respectively (Fig. S5 dotted grey line, ESI†). However, after the wireless electrolysis, a peak at higher retention times (around 11 min) appears, associated with LAN-S, corroborating LAN enantiomers' wireless reduction (Fig. S5 black line, ESI†). However, under these conditions, a relatively low apparent product yield (%PI_app_) was obtained (18.3%). Interestingly, the LAN-SO signal, which seems close to the d-LAN peak during the HPLC analysis, is not observed. Thus, the oxidation at the feeder anodes produces overoxidized forms of LAN and their concomitant decomposition. Nonetheless, the produced LAN-S at the cathodic side of the BPE can be easily separated with a fraction collector coupled to the chiral HPLC, as depicted from the correspondent chromatogram (Fig. 1a). As expected, the obtained fraction is a mixture mainly containing LAN-S, with a relatively low amount of the enantiomers of LAN (Fig. 1a).

**Fig. 1:**
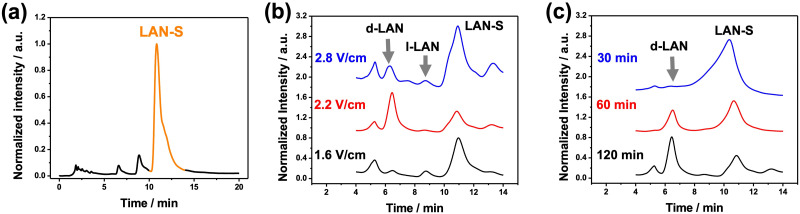
(a) Chromatogram of the fraction collector coupled to the chiral HPLC, obtained after the electroorganic reduction of LAN (10 mM) in a 5 mM LiClO_4_ ACN solution. Chromatograms of the product mixtures obtained for the wireless asymmetric synthesis of LAN as a function of (b) the applied *ε* (120 min) and (c) electrolysis time (*ε* = 2.2 V cm^−1^).

With the so-collected LAN-S, we performed the wireless enantioselective oxidation taking place at the oligomer/solution interphase. In this case, the 3 cm graphite rod was modified on one extremity with the oligo-(*R*)-BTIndT_4_. Once again, the BPE was immobilized by a silicon ring at the center of a closed bipolar cell containing a 5 mM LiClO_4_ ACN solution. Since the enantioselective oxidation occurs at the modified extremity of the BPE, this was placed facing the feeder cathode. The LAN-S obtained in the previous step was injected into the anodic chamber, and enantioselective oxidation was performed. At first, we compare the performance of our system by applying different *ε* values for a constant electrolysis time (120 min). As can be seen from the chromatograms, when using the oligo-(*R*)-BTIndT_4_ modified BPE, and by applying a low electric field (1.6 V cm^−1^), the peaks associated with the LAN enantiomers and LAN-S were observed (Fig. 1b, black line). Since the oligo-(*R*)-BTIndT_4_ exhibits a favorable diastereomeric interaction with the d-LAN, as demonstrated above, it is possible to assume that during the oxidation of LAN-S, the resulting solution will be enriched with the d-enantiomer. Hence, under the applied *ε* (1.6 V cm^−1^), the induced Δ*V* is not high enough to trigger the correspondent oxidation at the anode of the BPE. Thus, the composition of the solution remains unchanged. However, when applying an *ε* value of 2.2 V cm^−1^, the peak associated with d-LAN is predominant compared to the signals of l-LAN and LAN-S (Fig. 1b, red line). In contrast, at higher electric field values (2.8 V cm^−1^), the chromatogram shows a mixture of all the different redox states of LAN; both LAN enantiomers, the starting compound (LAN-S) and the oxidized form (LAN-SO) (Fig. 1b, blue line). The LAN-SO formation is associated with the high polarization potential difference induced by the applied electric field (2.8 V cm^−1^), which triggers the different oxidation steps of LAN. Moreover, the relatively low amount of LAN enantiomers depicted on the chromatogram can be associated with their concomitant reduction at the feeder cathode. This explains the increase in the intensity of the LAN-S signal on the chromatogram since the reduced form is continuously generated at the feeder cathode. Therefore, an electric field of 2.2 V cm^−1^ was chosen to evaluate the electrolysis time's influence on the reaction composition. As expected, under these conditions, a gradual increase of the chromatographic peak associated with d-LAN and a concomitant depletion of the LAN-S signal were observed as a function of time (Fig. 1c).

To provide a more quantitative analysis of the wireless asymmetric synthesis, the ee and the %PI_app_ were calculated for all the experimental conditions. As expected, at low electric field values, the ee and %PI_app_ remain constant at around 35% and 4%, respectively, considering the d-LAN during the whole electrolysis (Fig. 2a and Fig. S6, black line, ESI†). Interestingly, the enantioselectivity was retained after one hour of electrolysis for a higher electric field value (2.8 V cm^−1^). In contrast, at longer times (120 min), the l-LAN enantiomer is produced (ee ≈ 50%) (Fig. 2a, blue line). Unfortunately, under these conditions, relatively low %PI_app_ values were obtained (below 10%) (Fig. S6, blue dots, ESI†). However, under the optimal *ε* (2.2 V cm^−1^), high ee values (>95%) for the d-enantiomer along the total time of electrolysis were obtained (Fig. 2a, red line), corroborating the efficiency for the wireless transfer of chirality. In addition, the %PI_app_ gradually increases as a function of electrolysis time, reaching a maximum value of around 60% (Fig. S6, red dots, ESI†). After these sets of experiments, the high enantioselectivity of both configurations of the oligo-BTIndT_4_ was tested. This was done by performing the enantioselective synthesis under the optimal experimental conditions (2.2 V cm^−1^, 120 min) with the oligo-(*S*)-BTIndT_4_ modified BPE. In this case, the formation of the l-LAN antipode is favored. As expected, when the wireless synthesis is carried out with the oligo-(*S*)-BTIndT_4_, the solution is enriched with the l-LAN enantiomer, whereas specular results were obtained when using the oligo-(*R*)-BTIndT_4_ modified electrode (Fig. 2b). In addition, the high enantioselectivity is corroborated by the ee values (above 90%) obtained for both BPE configurations under the same experimental conditions (Fig. 2a green and red dot).

**Fig. 2:**
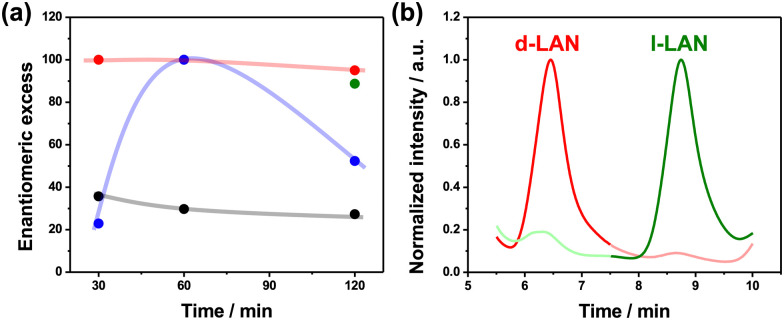
(a) Enantiomeric excess as a function of the time of electrolysis for different applied electric field values; 1.6 V cm^−1^ (black line), 2.2 V cm^−1^ (red line) and 2.8 V cm^−1^ (blue line) using an oligo-(*R*)-BTIndT_4_ modified BPE. The green dot represents the enantiomeric excess obtained using an oligo-(*S*)-BTIndT_4_ modified BPE. (b) Chromatograms of the product mixtures obtained for the wireless asymmetric synthesis of LAN using an oligo-(*R*)- (red line) or oligo-(*S*)-BTIndT_4_ (green line) modified BPE (*ε* = 2.2 V cm^−1^, 120 min).

Finally, the possible scale-up of the proposed umpolung approach was evaluated using a three-BPE array. At first, the electroreduction of a 10 mM LAN solution was performed by applying a constant *ε* value (2.2 V cm^−1^) for 120 min. As can be seen from the chromatograms, a considerable increase in the intensity of the signal related to LAN-S, with a steep decrease in the peaks of LAN, was obtained (Fig. S7, ESI†). This observation is significant compared to the single BPE system and is reflected in a substantial increase of the %PI_app_ from 18% to 80%. After this, the enantioselective synthesis under the optimal experimental conditions (2.2 V cm^−1^, 120 min) was carried out using a three oligo-(*S*)-BTIndT_4_ modified BPE array. Once again, the chromatogram reveals a single peak with a retention time close to 8 min, associated with the l-LAN, corroborating the enantioselectivity (Fig. S8, ESI†). As expected, a considerable increase of the %PI_app_ up to 95% for the BPE array was obtained, which is a significant enhancement compared to the single electrode device (≈ 60%).

In summary, the wireless umpolung transfer of chirality using inherently chiral oligomers has been demonstrated. The unique features of BE, in synergy with the enantioselective capabilities of the BTIndT_4_ oligomers, enable the efficient transfer of chirality even when starting from the racemic mixture of a given chiral analyte. The possible scale-up of the asymmetric conversion was demonstrated by using an array of multiple BPEs, increasing the %PI_app_ of the reaction from 60% up to 95%. Although the here proposed synthetic methodology presents all the usual intrinsic advantages of BE, such as its wireless nature, compared to classical electrosynthesis, this approach minimizes the use of supporting electrolytes. Furthermore, due to the asymmetric reactivity within a single object, it is possible to envision the study of electroorganic paired reaction pathways.^1^ However, to avoid contamination due to the formation of undesired side products on the feeder electrodes, the optimization of the bipolar electrochemical reactor *via* conducting membranes is required. Finally, the present approach opens up exciting perspectives for using inherently chiral oligomers as chiral environments for bulk asymmetric electrosynthesis of various electroactive chiral molecules.

This work has been funded by the H2020 European Research Council (ERC) under the HORIZON-ERC-2021 work program (grant agreement no. 101040798, ERC Starting grant CHEIR).

## Data availability

The data supporting this article have been included as part of the ESI.†

## Conflicts of interest

There are no conflicts to declare.

## Supplementary Material

CC-060-D4CC02406K-s001
