# Supplementary material for: Wireless asymmetric umpolung electrosynthesis
Source: Chem Commun (Camb). 2024 Jul 1;60(74):10120–3. doi: 10.1039/d4cc02406k (PMC11392033; doi:10.1039/d4cc02406k)
Supplement: CC-060-D4CC02406K-s001 [file CC-060-D4CC02406K-s001.pdf]

Electronic Supplementary Material (ESI) for ChemComm.
This journal is © The Royal Society of Chemistry 2024

Supporting Information

Wireless asymmetric umpolung electrosynthesis

Sara Grecchi,‡a Bartlomiej Bonczak,‡a Filippo Malacarne,a Gerardo Salinas,b Roberto Cirilli,c Serena Arnaboldi*a

aDip. Di Chimica, Univ. degli Studi di Milano, Milan, Italy.

bUniv. Bordeaux, CNRS, Bordeaux INP, ISM, UMR 5255, F-33607 Pessac, France

cIstituto Superiore di Sanità, Centro Nazionale per il Controllo e la Valutazione dei Farmaci, Rome, Italy.

‡These authors contribute equally

1) Materials and methods

Lithium perchlorate, $LiClO_4$ (Aldrich, ≥99.9%), Tetrabutylammonium perchlorate, TBAP (Aldrich, ≥95%), acetonitrile, ACN (Aldrich, ≥99.9% HPLC grade), racemic lansoprazole (Sigma-Aldrich), solvents for the HPLC (ACN, Aldrich, ≥99.9%, and water, CAS number: 7732-18-5) were used as received. The oligo-(R)- and oligo-(S)-BTIndT4 monomers were synthesized and separated following a sequence of reactions previously reported.[1-4]

1.1) Oligomerization of oligo-(R)-/(S)-BTIndT4 films and enantioselective test.

The potentiodynamic electrochemical oligomerization of BTIndT4 was carried out in a classic three-electrode mini cell (3 mL of solution) containing a 0.75 mM monomer, 0.1 M TBAP ACN solution. For this purpose, a 3 cm length graphite rod (Ø =0.3 cm), acting as a working electrode, coupled to a Pt and Ag wire as counter and reference electrodes, respectively, were used. All classic electrochemical measurements were performed using a PalmSense4 potentiostat connected to a personal computer. The enantiorecognition capability of the inherently chiral films toward (d)-lansoprazole was tested by using modified graphite working electrodes with either the oligo-(R)- or oligo-(S)-BTIndT4. The potentiodynamic analysis was conducted with a coiled Pt wire and Ag|AgCl (as counter and reference electrodes, respectively) in a 0.1 M $LiClO_4$ ACN solution containing 2 mM of (d)-Lans.

1.2) Wireless electroorganic synthesis.

All the bipolar electrochemistry experiments were carried out with a homemade closed bipolar electrochemical cell, maintaining a distance of 5 cm between the feeder electrodes. At first, the reduction of racemic lansoprazole was carried out in the cathodic chamber containing 3 mL of a 10 mM LAN, 5 mM $LiClO_4$ ACN solution using an unmodified 3 cm long graphite rod as BPE. In comparison, the anodic compartment contains exclusively the electrolytic solution (5 mM $LiClO_4$ in ACN). The cathodic chamber was deaerated with N_2 to reduce the formation of by-products. After 2 hours at a constant electric field of 2.2 V cm^{-1}, the cathodic solution was analyzed through HPLC to corroborate the LAN-S formation. This fraction was then collected through HPLC, stored, and used in the second part of the procedure. For the wireless transfer of chirality, an oligo-(R)- or oligo-(S)-BTIndT4 modified graphite rod was used as BPE (total length 3 cm). Such chiral BPE was used for the enantioselective oxidation of the collected LAN-S to an antipode of LAN at the anodic chamber. In this case, the cathodic compartment was filled with just the electrolyte solution. The enantioselective oxidation was performed by applying three different electric fields (2.2, 1.6, and 2.8 V cm^{-1}) and sampling the reaction mixture at three different times (after 30 min, 60 min, and 120 min).

1.3) High-Performance Liquid Chromatography (HPLC).

Chiral HPLC analyses were carried out with HPLC equipment (Agilent 1260 Infinity II) and a Daicel CHIRALPAK® IG-3 column in isocratic reverse phase conditions. The HPLC analyses of lansoprazole racemate, lansoprazole sulfide, and sulfone were performed by injecting 30 µL of each solution in the chiral column, with ACN/H_2O 55:45 as eluent and a 0.5 ml/min flow, T= 40°C. The photodiode array (PDA) detector was operating at a wavelength of 280 nm. To compare the results, the three compounds were tested separately and simultaneously under the same conditions.

1.4) Calculation of the apparent product yield.

The areas of each peak in the HPLC chromatogram were calculated with Origin® software. The three peaks recorded in each chromatogram are related to the precursor and the d- and l-enantiomers,

respectively. The apparent product yield (% PI) of each compound present in the sample was calculated as:

$$\%PI_{app} = \frac{Area\ of\ product}{Total\ Area}\ x\ 100$$

2) Electrooligomerization of BTIndT4 and enantioselective test of LAN enantiomers

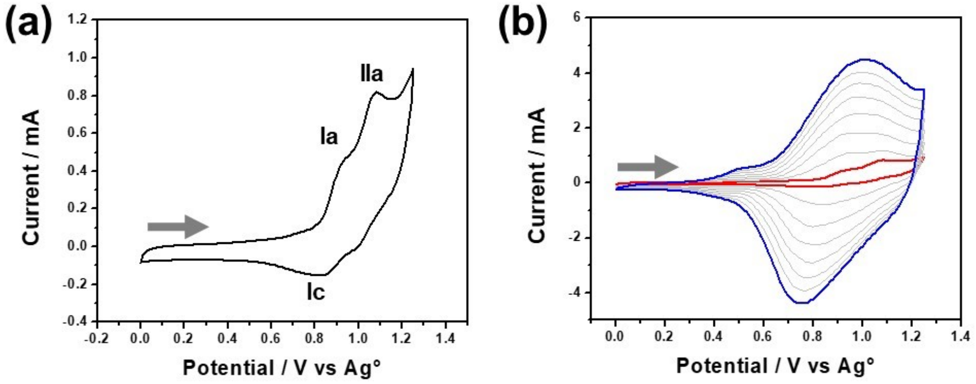

Figure S1 (a) Potentiodynamic oxidation of BTIndT4 obtained in a 0.75 mM monomer, 0.1 M LiClO4 ACN solution, graphite rod as working electrode, v = 0.2 V s-1. (b) Electrochemical oligomerization (72 cycles) of BTIndT4 obtained in a 0.75 mM monomer, 0.1 M LiClO4 ACN solution, graphite rod as working electrode, v = 0.2 V s-1.

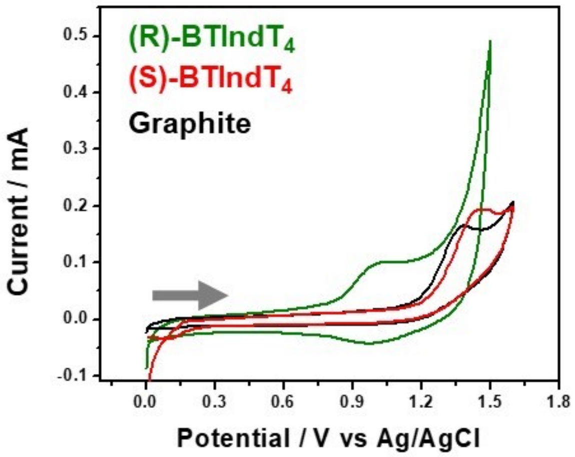

Figure S2 Enantioselective tests carried out in a 0.1 M LiClO4 ACN solution containing 2 mM d-LAN with oligo-(R)-BTIndT4 or oligo-(S)-BTIndT4 (indicated in the figure with green and red lines respectively), deposited on a graphite electrode. In black, the d-LAN signal was recorded on a bare graphite electrode.

3) Bipolar electrochemistry set-up

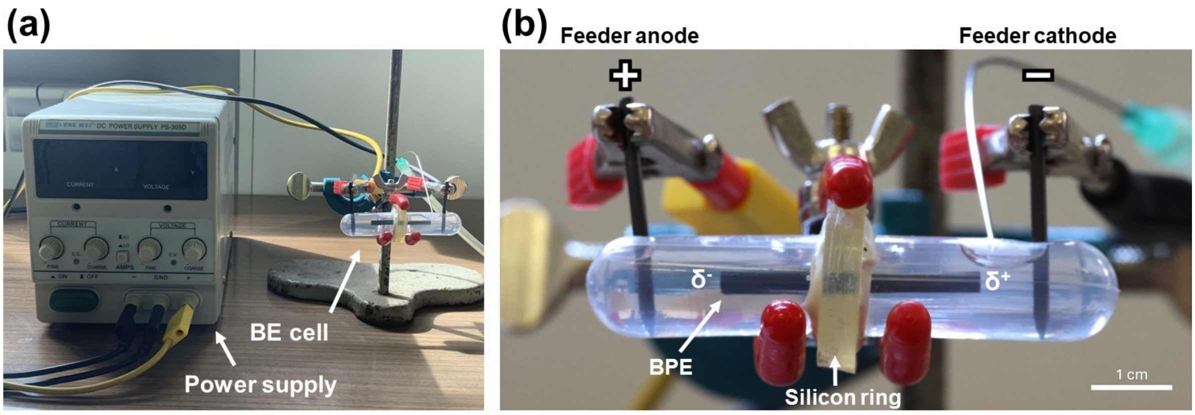

Figure S3 Optical pictures of (a) the complete bipolar electrochemistry set-up and (b) the compartmentalized bipolar electrochemical cell, showing the correspondent power supply, feeder electrodes, and bipolar electrode.

4) HPLC analyses

4.1) Standard analyses of LAN, LAN-S and LAN-SO

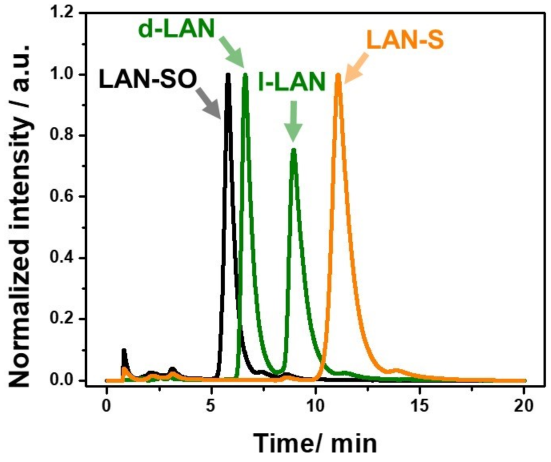

Figure S4 Chromatograms of LAN-SO (black line), LAN-S (orange line), and correspondent antipodes of LAN (green line).

4.2) Electroorganic reduction of LAN

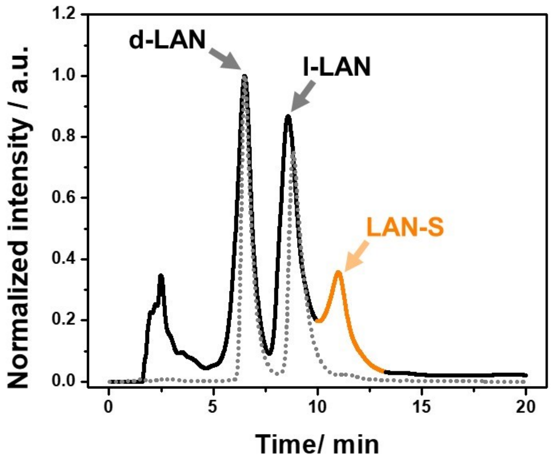

Figure S5 Chromatogram obtained before (grey dotted line) and after the electro-organic reduction (black line) of LAN (10 mM) in a 5 mM LiClO4 ACN solution. The orange line represents the characteristic signal of LAN-S.

4.3) Apparent product yield of the wireless enantioselective oxidation

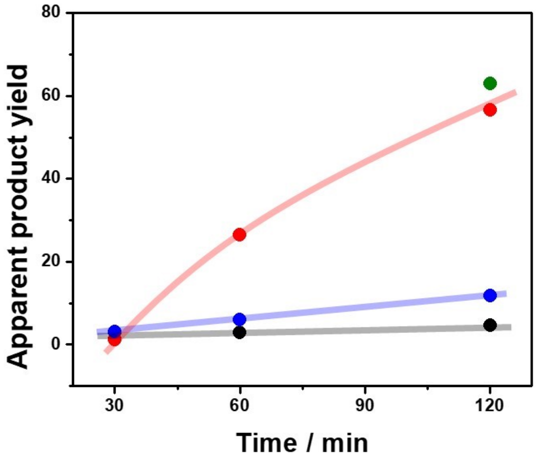

Figure S6 Apparent product yield as a function of the time of electrolysis for different applied electric field values: 1.6 V/cm (black line), 2.2 V/cm (red line), and 2.8 V/cm (blue line) using an oligo-(R)-BTIndT4 modified BPE. The green dot represents the product yield obtained using an oligo-(S)-BTIndT4 modified BPE.

4.4) Scale-up by using an array of multiple BPEs

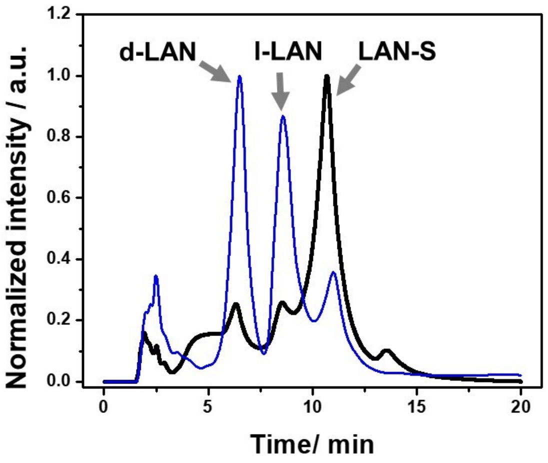

Figure S7 Chromatograms obtained for a single (blue line) and multiple BPE system (black line) after the electroorganic reduction of LAN (10 mM) in a 5 mM LiClO4 ACN solution.

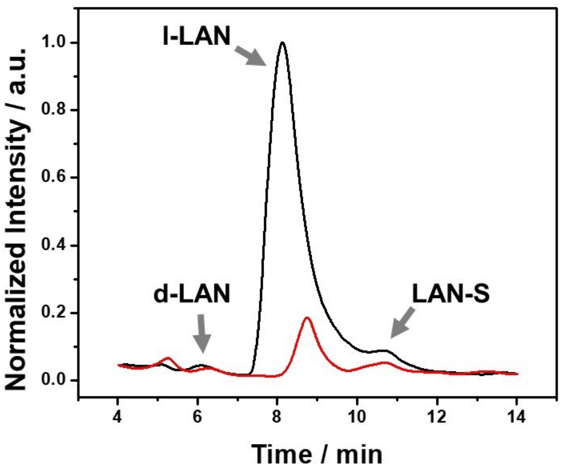

Figure S8 Chromatograms of the product mixtures obtained for the wireless asymmetric synthesis of LAN using a single (red line) or multiple (black line) oligo-(S)-BTIndT4 modified BPEs (ε =2.2 V/cm, 120 min).

References

V. Bocchi, G. Palla, Synthesis 1982, 12, 1096-1097.

Y. Zou, G. Yue, J. Xu, J. Zhou, Eur. J. Org. Chem. 2014, 27, 5901-5905.

J. S. Bair, R. G. Harrison, J. Org. Chem. 2007, 72, 6653-6661.

T. Zheng, J. Xu, S. Cheng, J. Ye, S. Ma, R. Tong, J. Org. Chem. 2023, 88, 11497-11503.
